# High-yield astaxanthin production process development and scale-up validation from wild-type *Phaffia rhodozyma* via parameter optimization and LSTM modeling

**DOI:** 10.3389/fmicb.2025.1667396

**Published:** 2025-08-29

**Authors:** Po Chen, Xingli Shi, Jingyan Jiang, Huanghe Cheng, Jinyan Chai, Zhenggang Xie, Mohd Helmi Sani

**Affiliations:** ^1^Gastroenterology and Urology Department II, Hunan Cancer Hospital/the Affiliated Cancer Hospital of Xiangya School of Medicine, Central South University, Changsha, China; ^2^Clinical Research Center for Gastrointestinal Cancer in Hunan Province, Changsha, China; ^3^T&J Bio-engineering (Shanghai) Co., Ltd., Shanghai, China; ^4^Department of Biosciences, Faculty of Sciences, University Technology Malaysia, Skudai, Johor, Malaysia

**Keywords:** *Phaffia rhodozyma*, astaxanthin, LSTM modeling, scale-up, fermentation optimization

## Abstract

**Introduction:**

This study developed an integrated strategy to significantly enhance astaxanthin production from wild-type *Phaffia rhodozyma* GDMCC 2.218, addressing the need for improved natural astaxanthin yields through non-genetically modified approaches.

**Methods:**

The research combined traditional parameter optimization with LSTM (Long Short-Term Memory) intelligent modeling. Systematic optimization of fermentation conditions was conducted in 500 mL bioreactors, followed by scale-up to 5 L systems. An innovative LSTM prediction model was constructed to predict astaxanthin concentration throughout the fermentation process.

**Results:**

Optimal fermentation conditions were determined as temperature 20°C, pH 4.5, and dissolved oxygen 20%, achieving an astaxanthin yield of 387.32 mg/L within 144 hours in 500 mL bioreactors. Upon scale-up to 5 L, the yield improved to 400.62 mg/L within 165 hours, demonstrating process robustness. The LSTM prediction model showed excellent performance with R^2^ = 0.978. The achieved yields represented a 10- to 20-fold improvement over previously reported wild-type strain levels and reached or surpassed the production levels of most engineered strains.

**Discussion:**

This research confirms the feasibility of achieving commercial-scale production of high-value natural astaxanthin through non-genetically modified approaches. The resulting product combines high productivity, safety, and regulatory advantages, providing an innovative solution for industrial-scale natural astaxanthin production that offers significant commercial potential.

## 1 Introduction

Astaxanthin-producing strains primarily encompass wild-type and engineered strains. Among wild-type strains, *Phaffia rhodozyma* (*P. rhodozyma*) and *Haematococcus pluvialis* represent key microorganisms for natural astaxanthin production. Compared to *H. pluvialis*, *P. rhodozyma* exhibits several advantages, including rapid heterotrophic metabolism that utilizes diverse sugars, shorter cultivation cycles, higher biomass utilization efficiency, and more industrially feasible fermentation processes (Gervasi et al., 2020; Nutakor et al., 2022; Guan et al., 2023). However, wild-type strains typically exhibit low product concentrations, presenting significant cost challenges for downstream separation and purification. The prevalent low-yield problem in wild-type strains, commonly reported at 1–30 mg/L levels in literature (Xiao et al., 2011; Mussagy et al., 2022; Lin, 2024), not only constrains production costs but also highlights the necessity of enhancing fermentation efficiency through process innovation. Engineered strains primarily include *Escherichia coli*, *Saccharomyces cerevisiae*, *Yarrowia lipolytica*, and genetically modified *P. rhodozyma*. These strains can improve astaxanthin fermentation efficiency and yield, attracting considerable research attention both domestically and internationally. Reports indicate that engineered strains can achieve final yields of tens to hundreds of milligrams per liter (Park et al., 2018; Zhou et al., 2019). However, products from engineered strains require strict regulatory oversight to ensure that no environmental or health risks are present. In contrast, wild-type strain (non-genetically modified organism) products, due to their higher safety profile, typically achieve easier regulatory approval and consumer acceptance (Zhang et al., 2020).

Traditional fermentation optimization primarily relies on parameter regulation (temperature, pH, dissolved oxygen, etc.) and culture medium composition adjustment (Villegas-Méndez et al., 2021). However, these approaches exhibit two significant limitations: first, single-factor or response surface methods struggle to capture multi-parameter dynamic coupling effects; second, experimental trial-and-error approaches incur high costs and difficulty in achieving real-time prediction. Culture medium composition and fermentation conditions represent critical factors affecting astaxanthin yield and production costs in *P. rhodozyma* fermentation. Cultivation temperature, pH, dissolved oxygen conditions, carbon-to-nitrogen ratio, carbon source composition, and nitrogen source composition all significantly influence astaxanthin production (Villegas-Méndez et al., 2021). Therefore, this study selected wild-type *P. rhodozyma* as the research subject.

In recent years, deep learning has provided new insights for overcoming traditional optimization bottlenecks (Tavasoli et al., 2019; Wang et al., 2021). Long Short-Term Memory (LSTM) networks, due to their exceptional temporal feature extraction capabilities, have demonstrated precise predictive performance in modeling fermentation processes, such as Monascus pigment, penicillin, and ethanol production (Sousa et al., 2021; Sun et al., 2023; Huang et al., 2024). However, intelligent modeling research specifically targeting *P. rhodozyma* astaxanthin fermentation remains relatively unexplored. This study innovatively combines LSTM neural networks with traditional parameter optimization methods: systematically optimizing key fermentation parameters to enhance baseline yield while establishing LSTM-based multi-parameter dynamic prediction models to analyze the temporal coupling patterns among pH, temperature, dissolved oxygen (DO), and biomass (wet weight). This dual-track strategy of “experimental optimization-data modeling” ensures process scalability (based on physical parameter control) while achieving real-time fermentation process prediction through data-driven approaches, providing a new paradigm for intelligent upgrading of natural astaxanthin production.

## 2 Materials and methods

### 2.1 Culture medium

All reagents in the following media were purchased from China National Pharmaceutical Group Chemical Reagents Co., Ltd. The composition of the medium and the sterilization treatment are as follows. Solid agar medium: Yeast extract (10 g/L), peptone (20 g/L), glucose (20 g/L), agar powder (20 g/L). Autoclaved at 121°C for 20 min and cooled to 40–50°C to prepare solid plates. Seed Medium (Optimized in the Laboratory): Yeast extract (10 g/L), peptone (20 g/L), glucose (20 g/L), KH₂PO₄ (3 g/L), MgSO₄·7H₂O (1 g/L). Autoclaved at 121°C for 20 min. Fermentation and Feeding Medium (Optimized in the Laboratory): Yeast extract (10 g/L), peptone (20 g/L), glucose (20 g/L), KH₂PO₄ (3 g/L), MgSO₄·7H₂O (1 g/L). Autoclaved at 121°C for 20 min. Supplement Feed Medium: Glucose (500 g/L), MgSO₄·7H₂O (15 g/L). Autoclaved at 121°C for 20 min.

### 2.2 Preparation of seed culture in shake flasks

The *P. rhodozyma* strain (GDMCC 2.218, Guangdong Microbial Culture Collection Center), stored at −80°C, was streaked on solid agar plates and incubated in a 20°C incubator for 2–3 days until clear red colonies were visible. A 100 mL seed medium was placed in a 500 mL baffled Erlenmeyer flask, sterilized, and cooled to room temperature. One loop of the colony was picked from the solid plate and inoculated into a flask. The flask was then placed on a shaker at 220 rpm and 20°C for 2–3 days.

### 2.3 Cultivation in bioreactors

The experimental design involved fermentation in 500 mL (CloudReady, T&J Bio-engineering.) and 5 L bioreactors (Intelli-Ferm A, T&J Bio-engineering), with the parameters set in Table 1. A batch-feeding strategy was adopted, and as the biomass grew, oxygen consumption increased. To ensure optimal oxygen supply, the stirring speed was continuously improved. If a downward trend in stirring speed was observed, it indicated nutrient depletion, prompting feeding control.

**Table 1:** Parameters for cultivation of *Phaffia rhodozyma.*

Parameter	500 mL	5 L
Initial volume (mL)	200	2,000
Stirring speed (rpm)	300–1,200	300–1,200
Aeration rate (VVM)	0.5	0.5
Inoculation volume	5%	5%
Feeding control (Constant rate)	0.8 mL/h	16 mL/h

### 2.4 Process optimization experiments in the 500 mL bioreactor

After confirming that the seed culture was free from contamination under the microscope, a 5% inoculum was introduced into the bioreactor for fermentation. The CloudReady 500 mL bioreactors were used for optimization experiments, focusing on temperature, pH, and DO gradients. For all three groups, agitation speed was maintained at 300–1,200 rpm (positively cascaded with dissolved oxygen), aeration rate at 0.5 vvm, and feeding was initiated at 0.8 mL/h when the agitation speed began to decrease continuously.

*Effect of temperature gradient on astaxanthin production* According to the parameters in Table 1, the influence of different fermentation temperatures on astaxanthin production by *P. rhodozyma* was investigated, with temperature gradients set at 20°C, 22°C, 25°C, and 28°C, pH 5.0, and DO 30%.

*Effect of pH gradient on astaxanthin production* Based on the temperature gradient optimization results, the influence of different fermentation pH values on astaxanthin production was assessed, with pH gradients set at 3.5, 4.0, 4.5, and 5.0. The temperature from the previous experiments was optimal, with DO set at 30%.

*Effect of DO gradient on astaxanthin production* Following the temperature and pH gradient optimizations, the influence of various DO levels on astaxanthin production was studied, with DO gradients set at 10, 20, 30, and 40%. The temperature and pH used were those determined to be optimal in previous experiments.

### 2.5 LSTM model construction and training

This study constructed an LSTM prediction model based on time-series data from 15 batches of 500 mL four-parallel bioreactor fermentation experiments. The original dataset encompassed four key process parameters: pH, dissolved oxygen (DO), temperature (temp), and wet weight (ww), along with the target variable astaxanthin concentration (AST). Four process parameters were acquired through online sensors, while the target variable was obtained via offline detection. Data preprocessing comprised three steps: (1) Batch identification—Regular expressions were used to match four input features and one target value corresponding to offline sampling time points across different fermentation batches. (2) Time series alignment—Since fermentation duration and sampling points varied between batches, each batch employed an independent time series as baseline, with variable-length sequences efficiently handled through mask synchronization, dynamic padding, and packing/unpacking mechanisms. (3) Standardization—Input variables underwent Z-score normalization to eliminate dimensional differences, as shown in the equation:


$$z=\frac{x-\mu }{\sigma }$$

Where *μ* and *σ* represent the mean and standard deviation of the four input variables (pH, DO, temperature, and wet weight) calculated from the training set.

Model architecture and hyperparameters were determined through grid search. The gating mechanism comprised Sigmoid functions (controlling information forgetting and updating) and tanh functions (regulating candidate memory cell states). The input layer received four-dimensional time series data (pH, DO, temp, and WW), while the output layer employed a linear activation function to predict astaxanthin concentration directly. The validation set was randomly split from the original dataset at a 10% ratio. During training, mean squared error (MSE) between predicted and experimental values served as the loss function, with Adam optimizer employed for parameter updates, and an early stopping mechanism implemented to prevent overfitting.

All modeling work was implemented using the PyTorch 2.7.1 framework, with a hardware platform comprising a 12th-generation Intel Core i7-1260P processor.

### 2.6 5 L bioreactor scale-up experiments and LSTM model validation

After confirming that the seed culture was free from contamination, a 5% inoculum was introduced into the bioreactor for fermentation. The 5 L bioreactor was employed for scale-up cultivation, adhering to the parameters outlined in Table 1 and utilizing the optimal temperature, pH, and DO determined from the 500 mL bioreactor optimization. To systematically evaluate process scalability, the LSTM prediction model established at 500 mL scale was applied to process monitoring of the 5 L reactor: pH, DO, and temperature data were collected in real-time through online sensors, biomass wet weight was determined by sampling, and the time-series data were subjected to standardization processing before being input into the pre-trained model, ultimately outputting predicted astaxanthin concentration trends. To validate the reliability of model prediction results, simultaneous offline sampling and determination of astaxanthin concentration were conducted.

### 2.7 Treatment of astaxanthin

After optimization, the procedure was as follows (Jiang et al., 2024). 1 mL of fermentation broth was placed in a 1.5/2 mL centrifuge tube and centrifuged at 9,660 g (MiniSpin® plus, Eppendorf AG, Germany) for 5 min to remove the supernatant. Then, 1 mL of 3 mol/L hydrochloric acid was added, followed by a boiling water bath for 4 min, rapid cooling, and another centrifugation at RCF 9,660 × g for 5 min to remove the supernatant. Subsequently, 1 mL of acetone was added for extraction for 30 min until the biomass was colorless, followed by centrifugation at RCF 9,660 × g for 5 min to retain the extract for HPLC (Waters Arc) analysis.

### 2.8 Analysis of astaxanthin by HPLC

Astaxanthin standard was obtained from Sigma-Aldrich (purity ≥ 97%). A standard curve was prepared with concentrations (20 mg/L, 40 mg/L, 60 mg/L, 80 mg/L, 100 mg/L). Liquid chromatography equipment: Waters, with the following chromatographic conditions: Waters XBridge® C_18_ column (4.6 × 50 mm); mobile phase: methanol–water (95:5); flow rate: 1 mL/min; detection wavelength: 475 nm (Lu et al., 2010).

### 2.9 OD_600_ measurement

OD_600_ measurement is based on the Beer–Lambert law, where the absorbance of a solution is proportional to the concentration of absorbing substances. Using a spectrophotometer (V-1100D Spectrophotometer, Shanghai Meipuda Instrument Co., Ltd.) set at 600 nm, the culture medium without bacteria was the blank for calibration. The bacterial culture was then measured in the spectrophotometer to obtain the OD_600_ value, which correlates with bacterial cell concentration.

### 2.10 Measurement of wet weight

An empty 1.5 mL/2 mL centrifuge tube was accurately weighed on an analytical balance to record its weight. Then, 1 mL of fermentation broth was added to the empty centrifuge tube and centrifuged at RCF 9,660 × g for 5 min to remove the supernatant. The wet weight of the biomass was determined by subtracting the weight of the empty tube from that of the tube containing the biomass.

### 2.11 Statistical analysis

Data are presented as mean ± standard deviation (SD) and statistical analysis was performed using Origin 2024.

## 3 Results

### 3.1 Temperature gradient optimization in the 500 mL bioreactor

**Figure 1:**
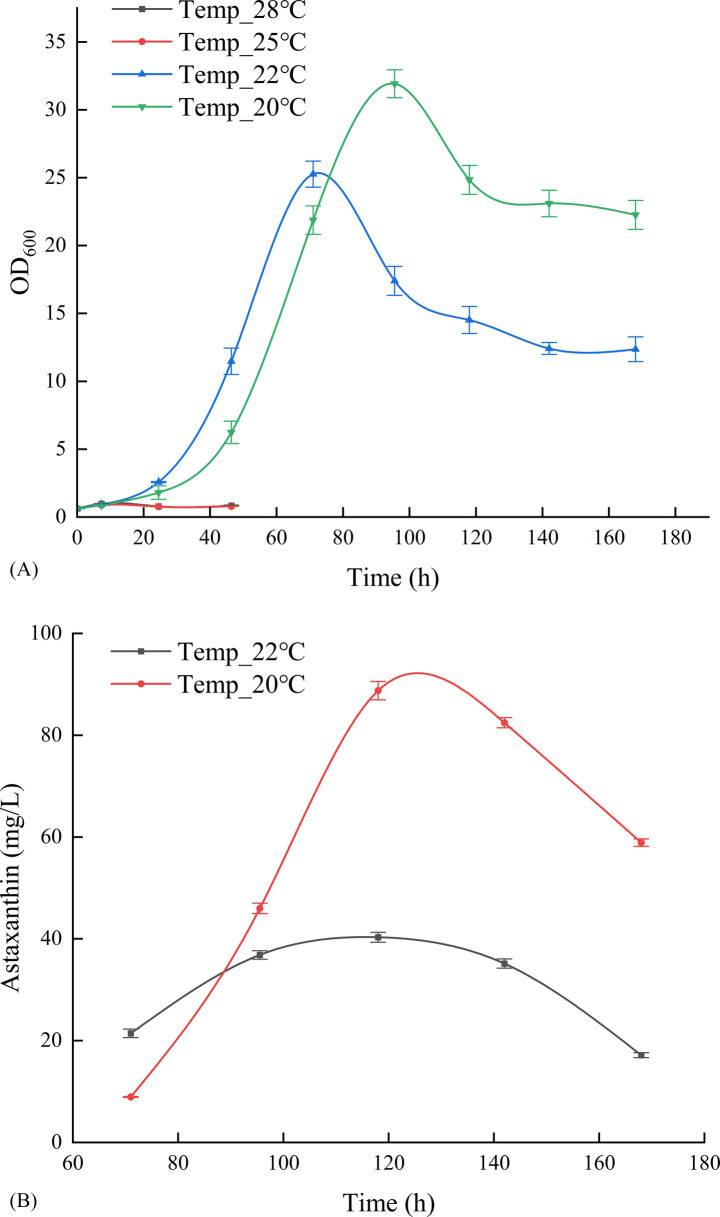
Effect of cultivation temperature on OD₆₀₀ **(A)** and astaxanthin production **(B)**.

As illustrated in Figure 1A, *P. rhodozyma* GDMCC 2.218 exhibited slow growth at fermentation temperatures of 25°C and 28°C, with an OD_600_ of less than one after 46.4 h, necessitating termination of cultivation for off-gassing treatment. Conversely, at 20°C and 22°C, the culture transitioned into a rapid growth phase following the lag period, continuing until 168 h before off-gassing. Figure 1B shows that the optimal result for the current strain was achieved at a fermentation temperature of 20°C, with an OD_600_ of 24.84 and an astaxanthin concentration of 88.76 mg/L after 118 h of fermentation. Thus, 20°C was most conducive to astaxanthin production among the tested temperature gradients by this strain.

### 3.2 pH gradient optimization in the 500 mL bioreactor

**Figure 2:**
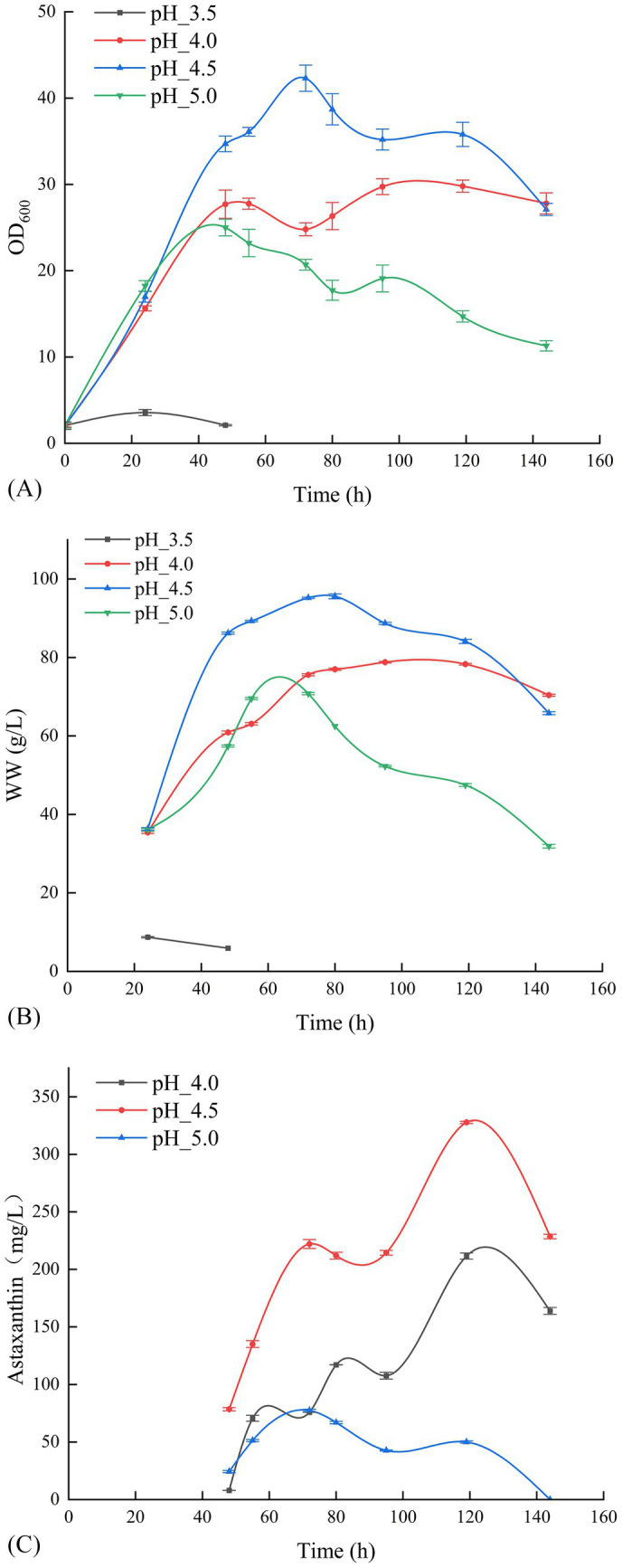
Effect of different pH levels on the OD_600_ **(A)**, the wet weight **(B)**, and the production of astaxanthin **(C)**.

Building on the temperature gradient optimization results, pH gradient optimization was conducted at the optimal fermentation temperature of 20°C and DO of 30%. Figure 2A illustrates that *P. rhodozyma* GDMCC 2.218 exhibited slow growth at a fermentation pH of 3.5, yielding an OD_600_ of only 2.1 after 48 h, leading to the termination of cultivation for off-gassing treatment. Other pH levels rapidly transitioned into the growth phase after overcoming the lag period, continuing until 144 h before off-gassing. Figure 2 indicate that the strain’s optimal result was achieved at a pH of 4.5, with an OD_600_ of 35.8 and a biomass wet weight of 84.1 g/L. This resulted in an astaxanthin concentration of 327.73 mg/L after 119 h of fermentation. Thus, pH 4.5 was found to be more favorable for astaxanthin production by this strain.

### 3.3 DO gradient optimization in the 500 mL bioreactor

**Figure 3:**
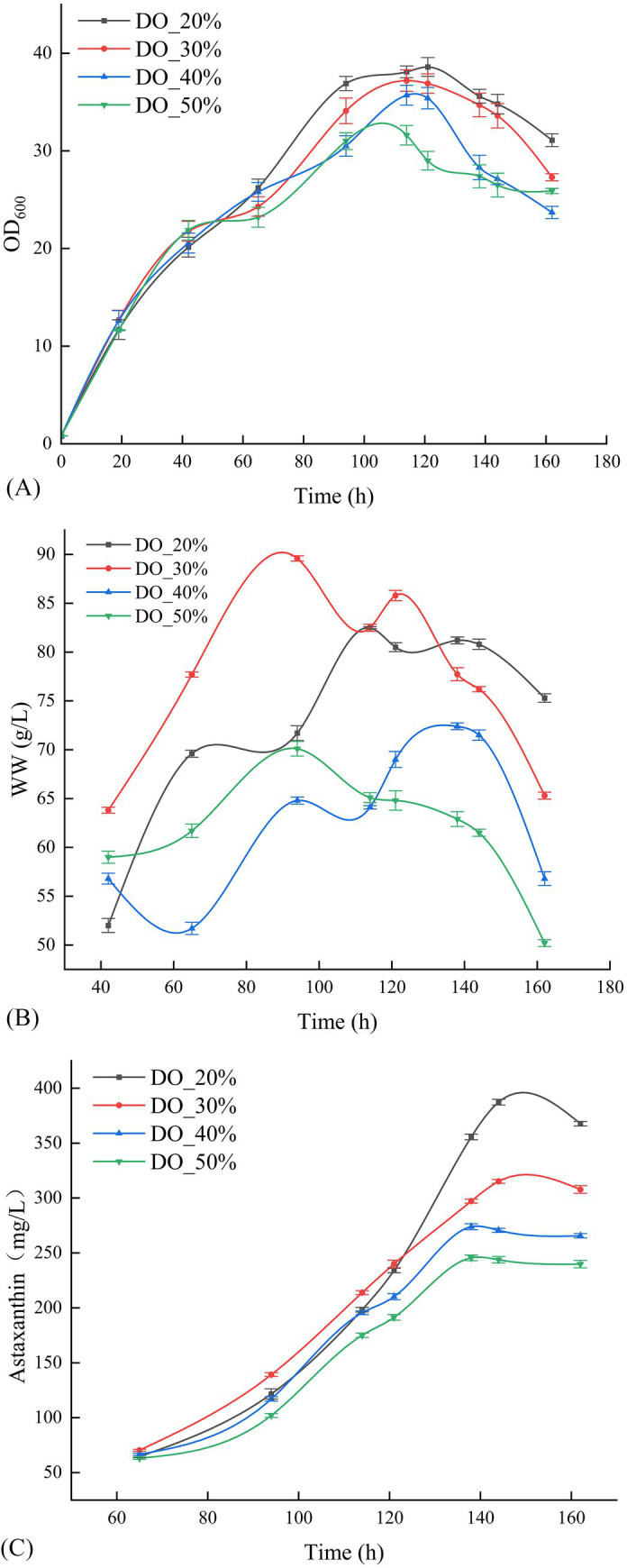
Effect of different DO levels on the OD_600_ **(A)**, the wet weight **(B)**, and the production of astaxanthin **(C)**.

Following the optimization of temperature and pH gradients, the optimal fermentation conditions were established at 20°C and a pH of 4.5. Figure 3 demonstrate that, compared to the temperature and pH gradient optimization experiments, *P. rhodozyma* GDMCC 2.218 swiftly transitioned through the lag phase into the growth phase across all DO treatments. The differences in astaxanthin yield post-fermentation were insignificant, indicating that DO levels had a less pronounced effect on astaxanthin production for this strain. Nonetheless, at DO 20%, the strain achieved the best results, with an OD_600_ of 34.8 and a biomass wet weight of 80.8 g/L, culminating in an astaxanthin concentration of 387.32 mg/L after 144 h of fermentation.

### 3.4 Training and validation of LSTM model on 500 mL fermentation data

The predictive performance of temporal models is highly dependent on hyperparameter configuration. To systematically determine the optimal parameter combination, this study employed grid search to evaluate different architectural performances with varying hidden unit numbers (32, 64, and 128). The three configurations achieved R^2^ values of 0.771, 0.978, and 0.882 on the training set, respectively (Supplementary Figure S1). Based on these results, a single-layer LSTM architecture was selected with 64 hidden units, Adam optimizer with a learning rate of 0.001, and a batch size of 4.

The LSTM model was constructed following the training methodology described above. Data preprocessing strictly adhered to fermentation process characteristics, with raw data processed through interpolation and forward/backward filling to ensure temporal continuity. After 4,058 iterations, the loss function gradually converged to its minimum value. An early stopping mechanism was triggered when validation loss showed no improvement for 1,000 consecutive epochs, with the model parameters corresponding to the minimum validation loss retained as the optimal configuration.

**Figure 4:**
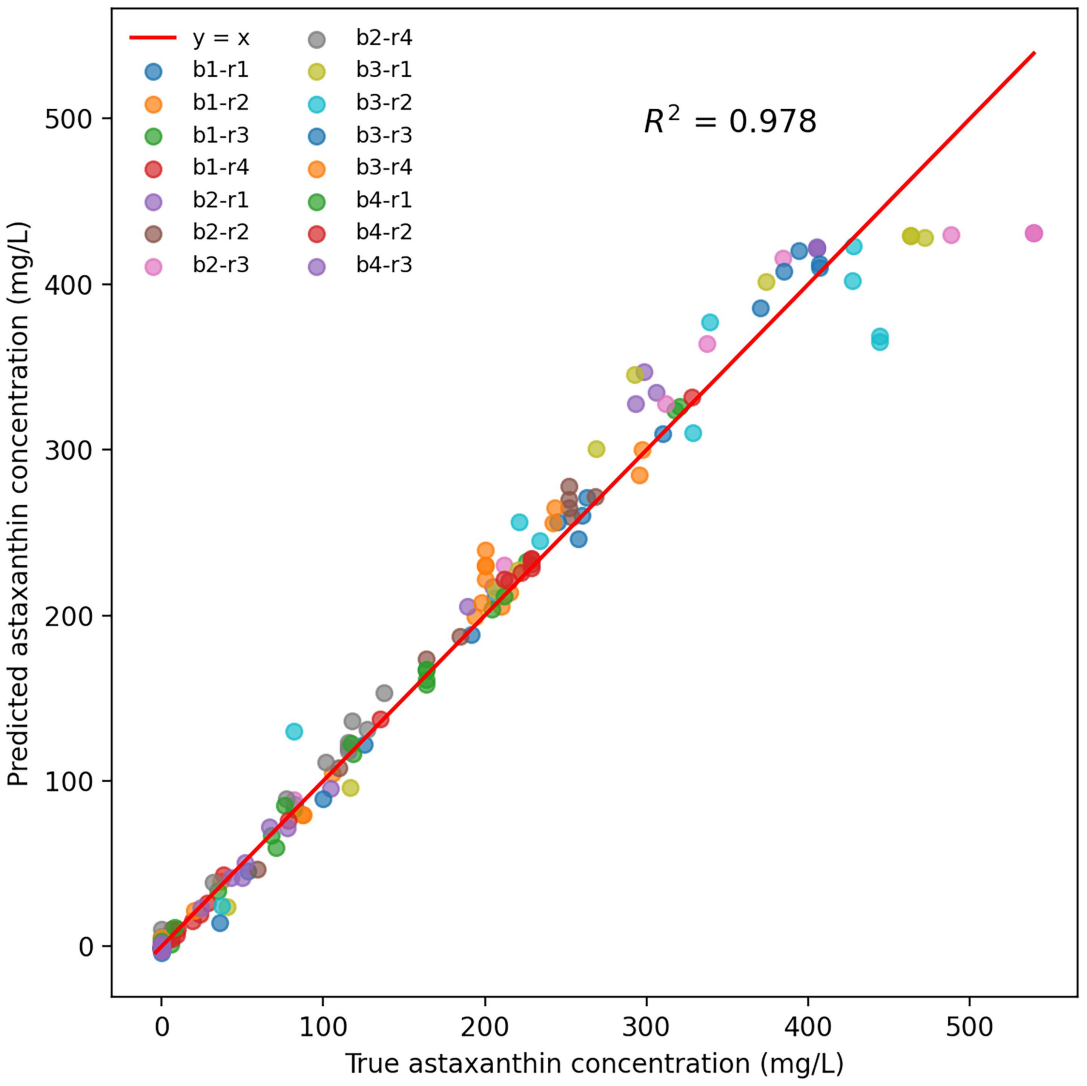
LSTM model prediction performance for astaxanthin concentration. b represents batch category, r represents reactor number. For example, b1-r1 represents reactor 1 of batch category 1.

The parameters corresponding to the minimum validation loss were retained as the optimal model. As shown in Figure 4, the predicted astaxanthin concentrations from 15 fermentation batches exhibited high concordance with measured values. The proximity of data points to the line y = x reflects minimal prediction error, with a correlation coefficient R^2^ of 0.978, demonstrating the LSTM model’s excellent regression performance on the training set. Notably, data points from different batches and reactors were closely distributed around the fitted line, indicating the model’s robustness to inter-reactor variability.

### 3.5 Process scale-up results and LSTM model predictions for 5 L bioreactor

**Figure 5:**
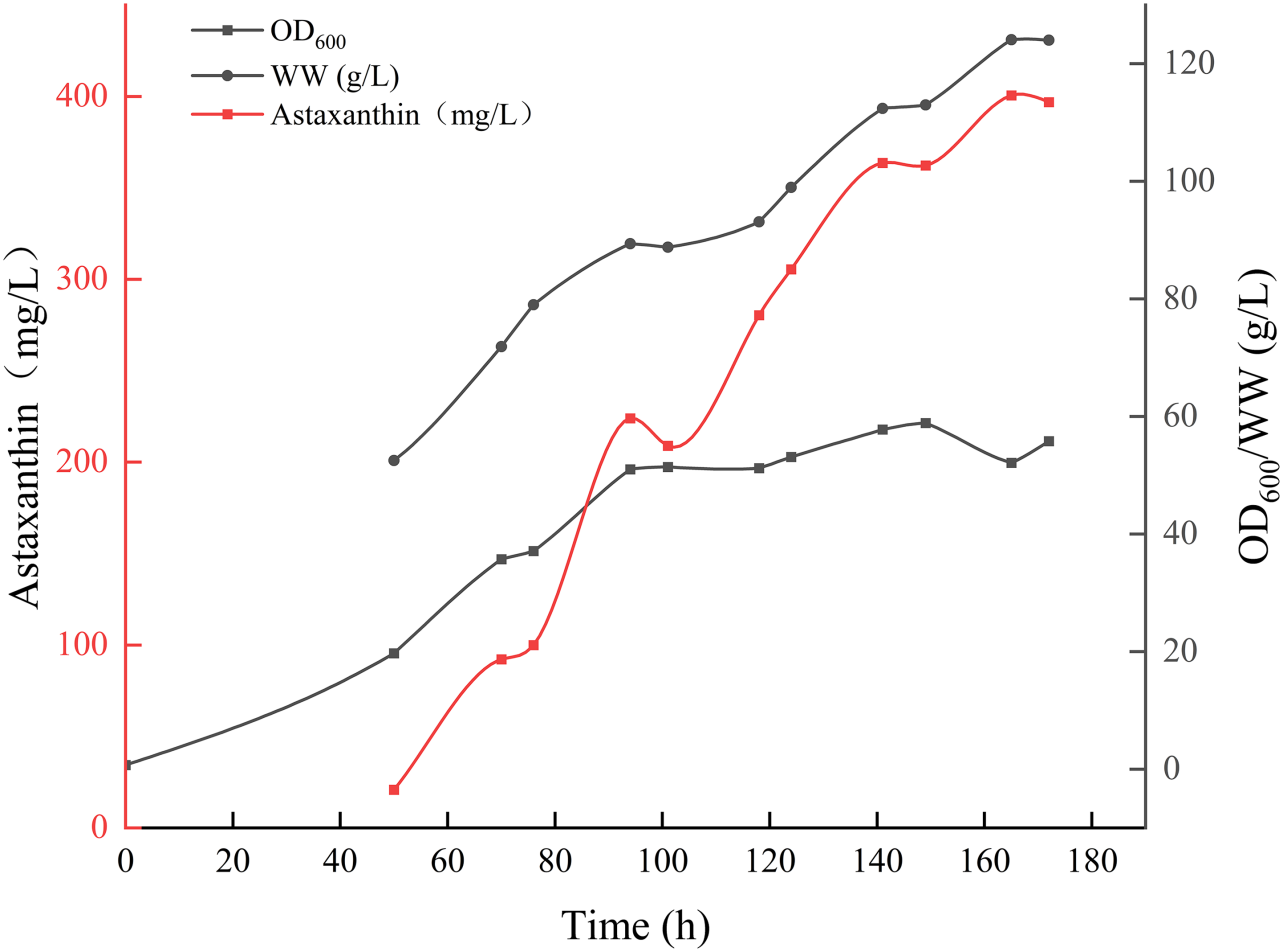
Time course of *Phaffia rhodozyma* growth and astaxanthin production in a 5 L bioreactor.

Based on the parameters outlined in Table 1, the optimal conditions, temperature 20°C, pH 4.5, and DO 20%, determined from the 500 mL bioreactor experiments, were implemented in the 5 L bioreactor for validation. Figure 5 indicates that this batch fermentation lasted 172 h, during which the astaxanthin yield gradually increased. It was observed that the yield slightly declined during the late growth stage. The optimal outcome was achieved after 165 h of fermentation, yielding an OD_600_ of 52.1, a biomass wet weight of 124.1 g/L, and an astaxanthin concentration of 400.62 mg/L. This indicates that the process effectively scaled up tenfold, achieving results that met or slightly exceeded those from the 500 mL bioreactor. The HPLC analysis spectra of the highest astaxanthin yield from the 5 L bioreactor (cultured for 165 h) and the standard (100 mg/L) demonstrated that the retention time of the astaxanthin peak in this analytical method was approximately 1 min, with a peak profile consistent with that of the standard (Supplementary Figure S2).

**Figure 6:**
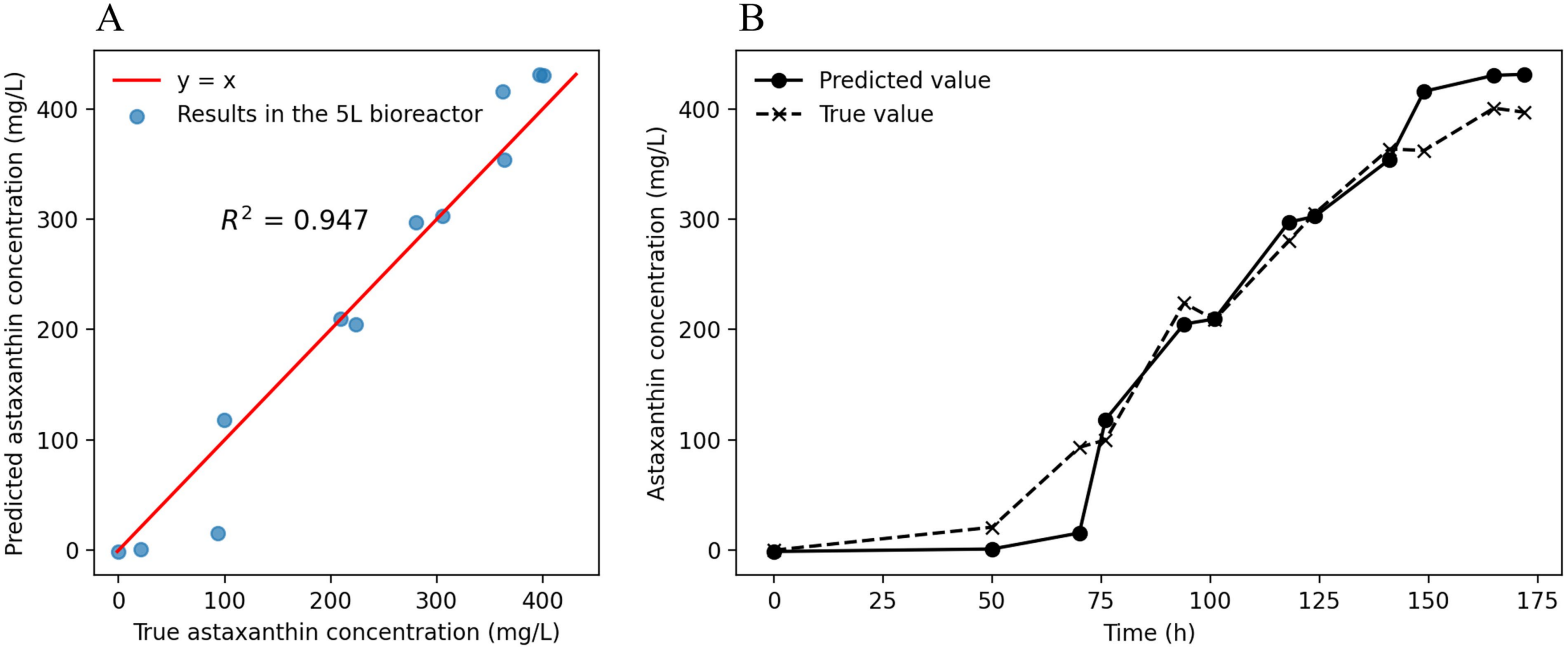
LSTM Model prediction results in 5 L bioreactor scale-up. **(A)** Fitting relationship between model-predicted values and measured values at a 5 L scale. **(B)** Temporal trends of predicted and measured astaxanthin concentrations at the 5 L scale.

The astaxanthin concentration prediction model, constructed based on fermentation experimental data from 15 batches at a 500 mL scale, was further applied to scale-up experiments in 5 L bioreactors for predictive validation, with the results shown in Figure 6. As demonstrated in Figure 6A, the model predictions showed high consistency with actual measured values at a 5 L scale, with the correlation coefficient (R^2^ = 0.947) indicating good generalization capability of the model for astaxanthin concentration prediction in bioreactors of different scales. From Figure 6, it can be observed that the predicted and measured astaxanthin concentrations showed high concordance in their trends over fermentation time, particularly in the mid-to-late fermentation phase (after 75 h), where the prediction curves accurately reflected the rising trend and dynamic changes of astaxanthin concentration, demonstrating the model’s effective learning of the nonlinear kinetic characteristics of astaxanthin synthesis.

## 4 Discussion

This study significantly enhanced astaxanthin production from wild-type *P. rhodozyma* GDMCC 2.218 through systematic optimization of fermentation parameters combined with LSTM modeling technology, providing important guidance for industrial-scale natural astaxanthin production. The results not only confirmed the effectiveness of traditional parameter optimization but also offered new insights for process scale-up through intelligent modeling techniques.

Regarding fermentation parameter optimization, temperature experiments demonstrated that 20°C was most favorable for astaxanthin synthesis, which aligns with the optimal growth characteristics of *P. rhodozyma* within the 17–21°C range. The yield reduction observed at high temperatures (25–28°C) may result from inhibition of DNA/RNA synthesis, reflecting the strain’s evolutionary adaptation to low-temperature environments (Miao et al., 2021; Shi et al., 2022; Mussagy et al., 2023). pH optimization results indicated that pH 4.5 represents the optimal condition for achieving the best balance between cell growth and astaxanthin synthesis. This weakly acidic environment maintains both cell membrane integrity and promotes metabolic enzyme function (Xie et al., 2014; Jia et al., 2024). Notably, this pH range also provides the natural advantage of inhibiting microbial contamination (Flores-Cotera et al., 2021; Mussagy et al., 2022), which is particularly important for industrial production. Dissolved oxygen experiments revealed that different DO levels (10–40%) had minimal impact on yield. However, yield was slightly higher at 20% DO, indicating that the GDMCC 2.218 strain possesses a robust oxygen utilization mechanism. This finding holds significant economic value, as maintaining high DO levels at industrial scale requires substantial energy consumption (Jahanian et al., 2024). Notably, during scale-up experiments from 500 mL to 5 L, the yield increased from 387.32 mg/L to 400.62 mg/L. This improvement may be attributed to enhanced mixing and oxygen transfer efficiency in larger vessels, while also validating the robustness of the optimized parameters.

Compared to conventional understanding, this study achieved a significant breakthrough: the obtained astaxanthin yields (387.32–400.62 mg/L) not only far exceeded reported wild-type strain levels (typically <50 mg/L) but even approached or surpassed the yields of some engineered strains (Park et al., 2018; Mussagy et al., 2022; Lin, 2024). This achievement demonstrates that through systematic parameter optimization rather than genetic modification, commercially competitive yields can be obtained while maintaining the safety and regulatory advantages of wild-type strains.

This study achieved systematic innovation in intelligent modeling for *Rhodotorula* yeast. While current research predominantly focuses on biomass and astaxanthin prediction in *Haematococcus pluvialis*—such as Cui et al., who developed an ANN (artificial neural network) model based on light and temperature (R^2^ > 0.98), and Liyanaarachchi et al., who created a carbon source optimization model (R^2^ > 0.91)—this work represents the first temporal process model for *Rhodotorula* yeast and innovatively achieves cross-scale LSTM predictions from 500 mL to 5 L bioreactors (Cui et al., 2019; Liyanaarachchi et al., 2020). LSTM networks, with their unique gating mechanisms and memory units, more effectively handle nonlinear temporal features in fermentation processes. Experimental results demonstrated excellent generalization performance (R^2^ = 0.978), accurately capturing dynamic astaxanthin accumulation patterns during mid-to-late fermentation stages.

However, LSTM models require substantial high-quality training data and may exhibit underfitting or overfitting risks, as evidenced by prediction deviations in the initial stage (0–100 mg/L). This phenomenon may be attributed to mass transfer differences during scale-up (Oldshue, 1966) and insufficient temporal resolution in training data. Nevertheless, prediction errors at critical production nodes were significantly reduced, validating the universality of temporal feature extraction for scale-up applications. These achievements demonstrate significant advantages in strain specificity, model advancement, and prediction accuracy, providing a reliable theoretical foundation and technical support for industrial-scale cultivation of *Rhodotorula* yeast.

The achievements of this study are primarily attributed to the following: precise optimization of key parameters through systematic experimentation, effective feeding strategies that maintain optimal nutritional levels, the appropriate selection of culture medium components that support both growth and astaxanthin synthesis, strict monitoring of process parameters during fermentation, and auxiliary prediction by the LSTM model. These factors synergistically contributed to substantial yield improvements. Future research could explore other parameters such as illumination and trace element supplementation, develop fed-batch strategies for large-scale production, analyze metabolic flux distribution under optimized conditions, improve LSTM model prediction performance in early fermentation phases, and optimize downstream processes to enhance astaxanthin recovery rates. Through further integration of multidisciplinary approaches, comprehensive optimization of wild-type *P. rhodozyma* astaxanthin production is anticipated.

## 5 Conclusion

This study achieved an astaxanthin yield of 400.62 mg/L from wild-type *Phaffia rhodozyma* GDMCC 2.218 through systematic optimization of fermentation parameters combined with LSTM intelligent modeling, representing a 1–2 order of magnitude improvement over literature values and outperforming most engineered strains. Process scale-up validation demonstrated the excellent transferability of this technology (R^2^ = 0.947 at the 5 L scale), with the LSTM model accurately capturing the fermentation kinetic characteristics. The research findings not only confirm that non-genetically modified approaches can achieve commercial-scale yields, but their regulatory-friendly nature better aligns with current stringent requirements for natural products in the food and pharmaceutical industries, providing a reliable technological paradigm for industrial-scale natural astaxanthin production.

## Data Availability

The original contributions presented in the study are included in the article/Supplementary material, further inquiries can be directed to the corresponding authors.
