# Supplementary material for: High-yield astaxanthin production process development and scale-up validation from wild-type Phaffia rhodozyma via parameter optimization and LSTM modeling
Source: Front Microbiol. 2025 Aug 29;16:1667396. doi: 10.3389/fmicb.2025.1667396 (PMC12425955; doi:10.3389/fmicb.2025.1667396)
Supplement: Supplementary file 1 [file Supplementary_file_1.docx]

**High-Yield Astaxanthin Production Process Development and Scale-up Validation from Wild-Type *Phaffia rhodozyma* via Parameter Optimization and LSTM Modeling**

*Po Chen^1,2^, Xingli Shi^3^, Jingyan Jiang ^3^, Huanghe Cheng^3^, Jinyan Chai^3^, Zhenggang Xie^*3,4^ and Mohd Helmi San^*4^*

**Address:**

^1^ Gastroenterology and Urology Department Ⅱ, Hunan Cancer Hospital/the Affiliated Cancer Hospital of Xiangya School of Medicine, Central South University, Changsha 410013, China

^2^ Clinical Research Center for Gastrointestinal Cancer in Hunan Province, Changsha 410013, China

^3^ T&J Bio-engineering (Shanghai) Co., Ltd., No. 8 Mingnan Road, Songjiang District, Shanghai 201613, China

^4^ Department of Biosciences, Faculty of Sciences, University Technology Malaysia, Johor 81310, Malaysia

*Corresponding author：

Zhenggang Xie

[xiezhenggang@graduate.utm.my](mailto:xiezhenggang@graduate.utm.my)

Mohd Helmi Sani

helmisani@utm.my

Po Chen^1,2^ and Xingli Shi^3^ contributed equally to this work and share first authorship.


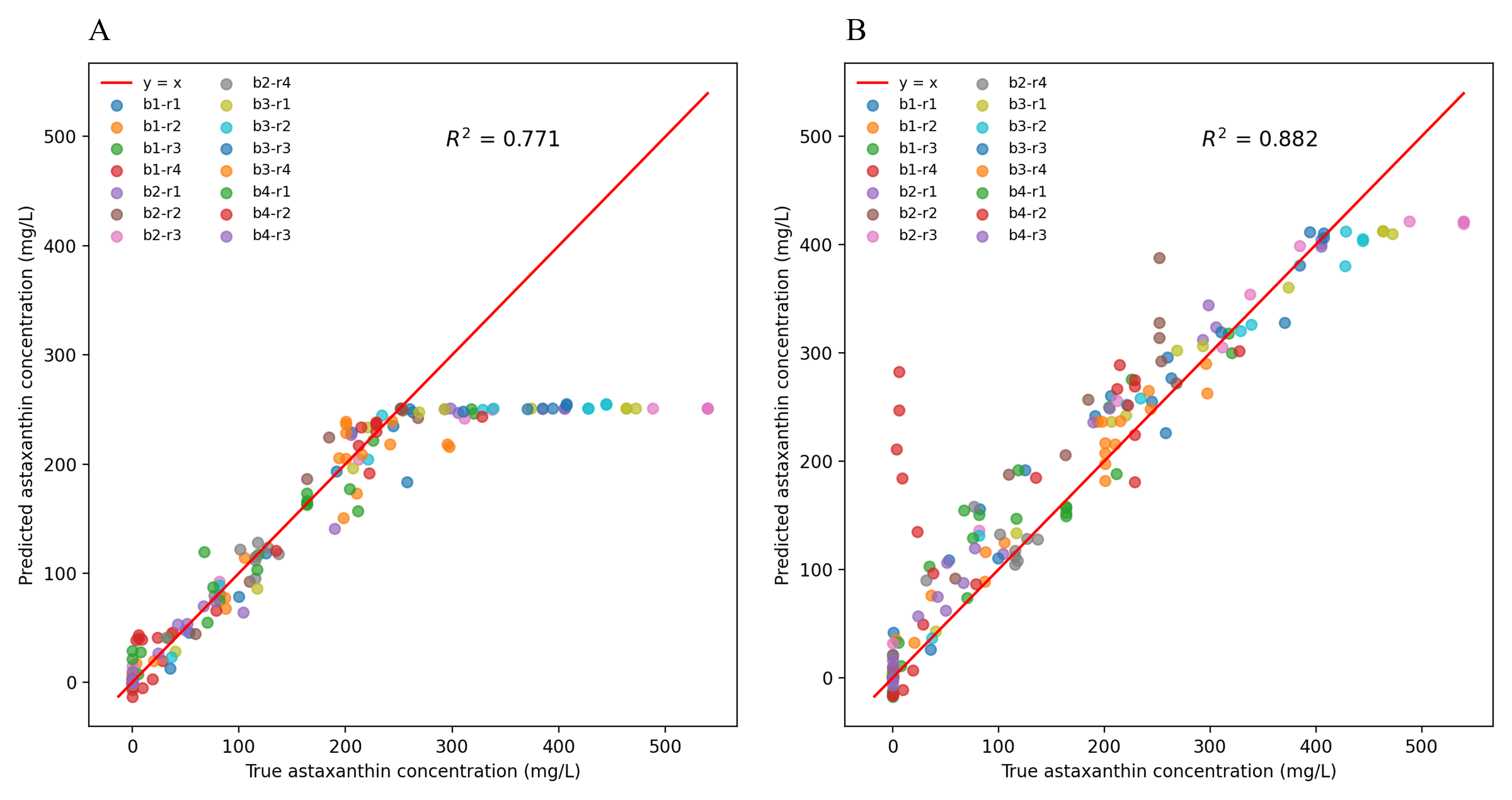


Figure S1 Training performance of LSTM models with different architectures

1. Number of hidden layer units is 32. (B) Number of hidden layer units is 128.
2.
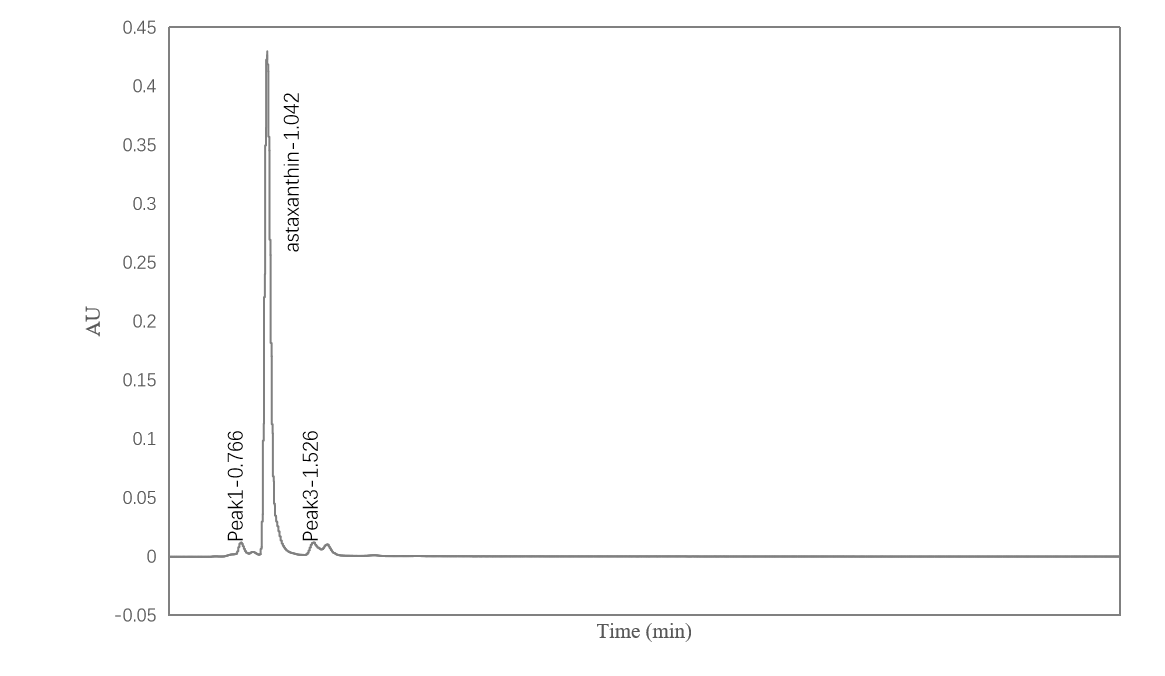

3.
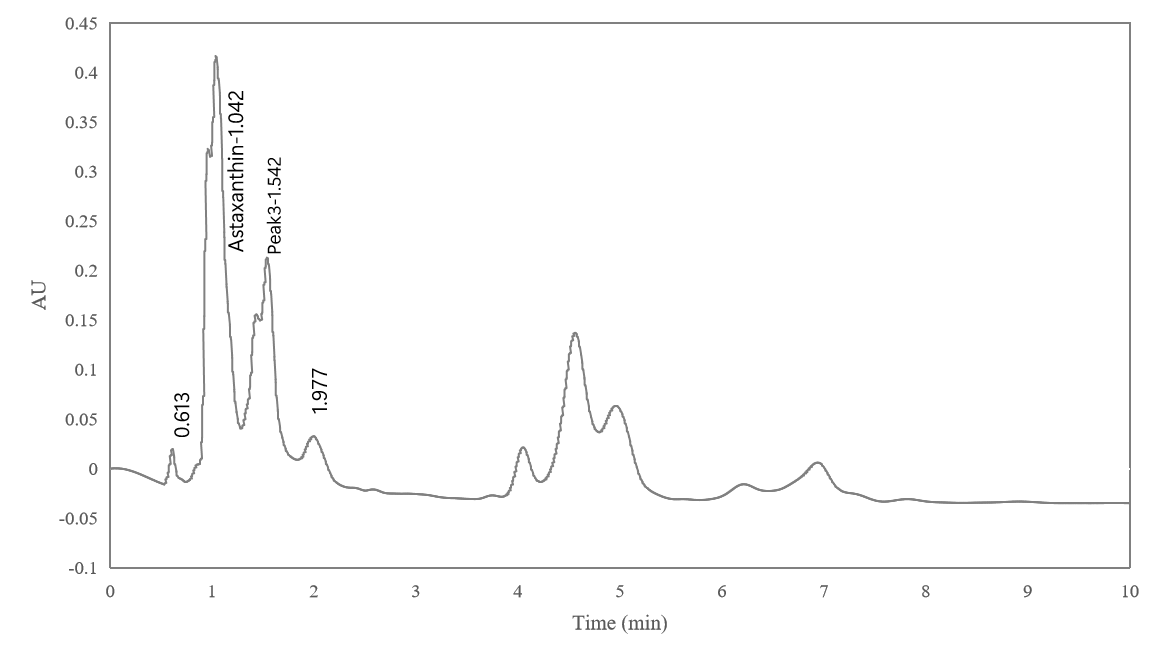


Figure S2 HPLC Analysis Spectrum of Astaxanthin for (A) Standard (100 mg/L) and (B) 5L Bioreactor (165h).
